# Laparoscopic *Versus* Open Surgery for Early-Stage Intrahepatic Cholangiocarcinoma After Mastering the Learning Curve: A Multicenter Data-Based Matched Study

**DOI:** 10.3389/fonc.2021.742544

**Published:** 2022-01-07

**Authors:** Yang Jinhuan, Wang Yi, Zheng Yuanwen, Ma Delin, Chen Xiaotian, Wang Yan, Deng Liming, Yu Haitao, Wu Lijun, Deng Tuo, Chen Kaiyu, Hu Jiawei, Zheng Chongming, Wang Daojie, Jin Bin, Chen Gang

**Affiliations:** ^1^ Department of Hepatobiliary Surgery, The First Affiliated Hospital of Wenzhou Medical University, Wenzhou, China; ^2^ Department of Epidemiology and Biostatistics, School of Public Health and Management, Wenzhou Medical University, Wenzhou, China; ^3^ Department of Hepatobiliary Surgery, Shandong Provincial Hospital, Jinan, China; ^4^ Department of Hepatobiliary Surgery, Qilu Hospital of Shandong University, Jinan, China; ^5^ Department of Clinical Medicine, The Second School of Medicine, Wenzhou Medical University, Wenzhou, China

**Keywords:** intrahepatic cholangiocarcinoma, laparoscopic hepatectomy, liver resection, overall survival, recurrence-free survival

## Abstract

**Background:**

Surgical resection is the only widely accepted curative method for intrahepatic cholangiocarcinoma (ICC). However, little is known about the efficacy of laparoscopic liver resection for ICC, especially in patients with early-stage disease. The aim of this study was to compare the short-term and long-term effects of laparoscopy and open surgery for the treatment of ICC.

**Methods:**

Data from 1,084 patients treated at three hospitals from January 2011 to December 2018 were selected and analyzed. Propensity score matching was performed to compare the long-term outcomes (overall survival and recurrence-free survival) and short-term outcomes (perioperative outcomes) of all-stage and early-stage patients.

**Results:**

After matching, 244 patients (122 *vs.* 122) in the all-stage group and 65 patients (27 *vs.* 38) in the early-stage group were included. The baseline of the two groups was balanced, and no significant differences were found in sex or age. The short-term results of the laparoscopic group were better than those of the open group, including less blood loss [blood loss ≥400 ml 27 (22.1%) *vs.* 6 (4.92%), p<0.001 for all-stage, 12 (31.6%) *vs.* 2 (7.41%), p=0.042 for early stage), shorter surgery [200 (141; 249) min *vs.* 125 (115; 222) min, p=0.025 for early stage] and shorter hospital stay [11.0 (9.00; 16.0) days *vs.* 9.00 (7.00; 12.0) days, p=0.001 for all stage, 11.0 (8.50; 17.8) days *vs.* 9.00 (6.50; 11.0) days, p=0.011 for early stage]. Regarding long-term outcomes, no significant differences were found for all-stage patients, while there were significant differences observed for the early-stage group (p=0.013 for OS, p=0.014 for RFS). For the early-stage patients, the 1-, 3-, and 5-year OS rates of the OLR group were 84.2, 65.8, and 41.1%, respectively, and those of the LLR group were 100, 90.9, and 90.9%, respectively. The RFS rates of the OLR group were 84.2, 66.7, and 41.7%, respectively, and those of the LLR group were and 92.3, 92.3, and 92.3%, respectively.

**Conclusion:**

Patients treated with laparoscopy seemed to have better short-term outcomes, such as less blood loss, shorter operation duration, and shorter hospital stay, than patients undergoing open surgery. Based on the long-term results, laparoscopic treatment for early ICC may have certain advantages.

## Introduction

Laparoscopic liver resection (LLR) has become a widely accepted surgical method (1–3) with equivalent safety and effectiveness to open liver resection (OLR) (3–6). Both minor and “difficult” major LLR procedures performed in large hepatobiliary centers have acceptable short-term and long-term outcomes (2, 7, 8). However, this conclusion is supported by studies related to hepatocellular carcinoma (HCC), benign tumors, or colorectal liver metastases (5, 9–11). Whether intrahepatic cholangiocarcinoma (ICC) is suitable for laparoscopic resection and the oncological outcome of laparoscopic resection for ICC are still unclear.

Randomized controlled trials (RCTs) are the gold standard for clinical studies, but they are difficult to implement in cancer-related surgical research due to uncontrollable factors such as tumor staging and differentiation. Propensity score (PS) analysis is a well-performed approach to estimate the causal treatment effects of clinical problems found in observational studies. The data generated from a large observational cohort (12, 13) can be used to evaluate important clinical problems when randomized trials are limited or non-existent.

Some articles have compared the short-term and long-term outcomes of LLR and OLR for the treatment of ICC (14–17), but no articles have explored the effect of laparoscopic hepatectomy for early-stage intrahepatic cholangiocarcinoma. The purpose of this study was to compare the short-term and long-term outcomes of LLR and OLR for the treatment of ICC, especially early-stage patients, to fully investigate the advantages and disadvantages of LLR and OLR among different patient populations.

## Methods

**Figure 1:**
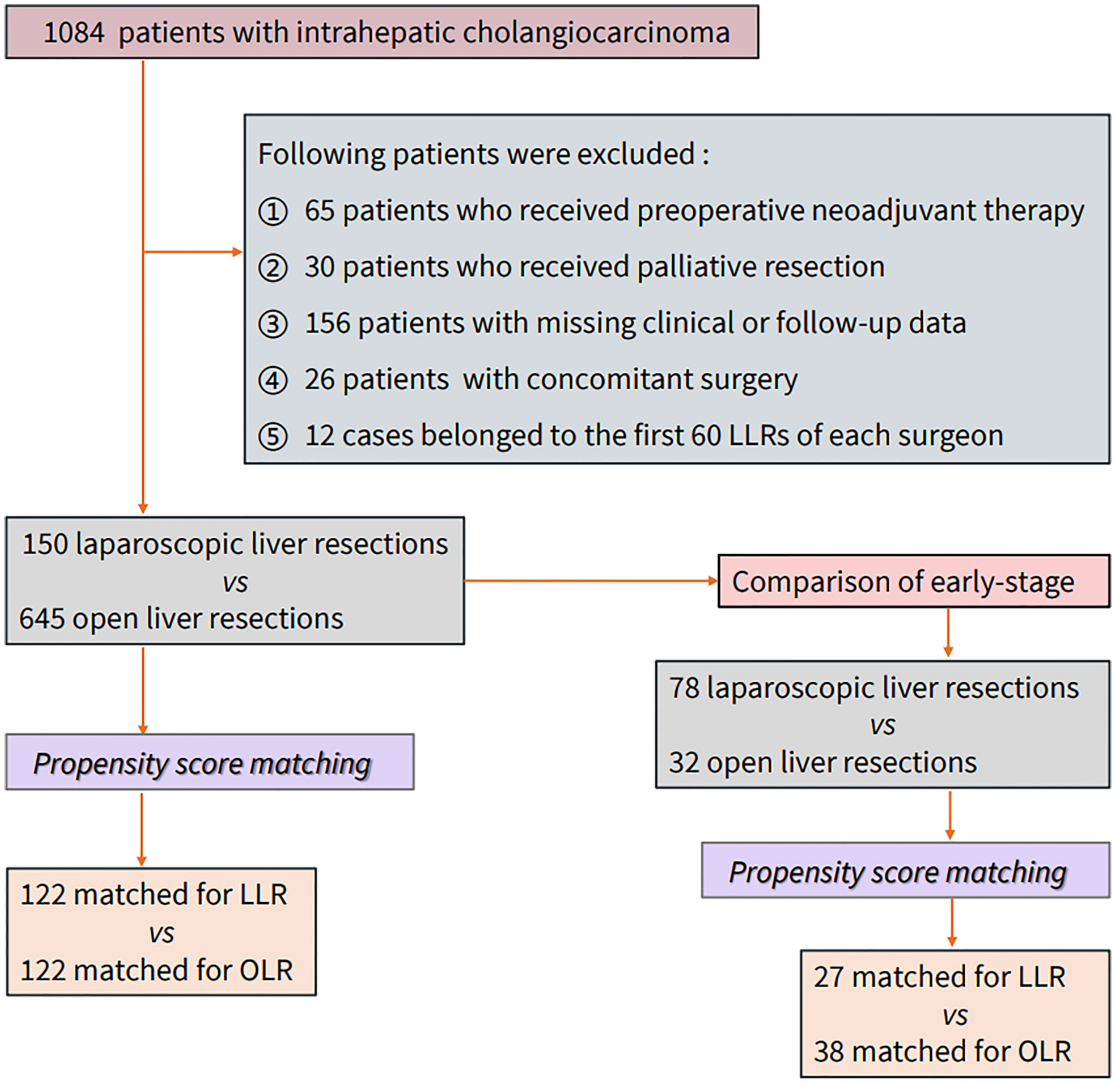
Research flow chart.

Patients diagnosed with ICC at 12 hepatobiliary surgical wards across three large hepatobiliary centers in southern and northern China from January 2011 to December 2018 were selected. Patients who underwent preoperative neoadjuvant therapy, palliative resection, or concomitant surgery and those with missing clinical or follow-up data along with cases of laparoscopic surgery early on in the mastery of the learning curve were excluded. According to different surgical methods, cases were divided into an open liver resection (OLR) group and a laparoscopic liver resection (LLR) group. Allocation to the LLR group was based on treatment intent. All operations selected were performed by senior hepatobiliary surgeons after mastering the learning curve (with at least 5 years of experience and ≥60 cases of LLR). Propensity score matching was used to reduce confounding factors and to promote a balance in the baseline characteristics. The matching factors were stratified according to the literature and clinical experience, including basic demographic information (sex, age, BMI, and smoking and drinking status), tumor pathology information (tumor size, tumor number, TNM stage, differentiation, lymphatic invasion, vascular invasion, and nerve invasion), and other important clinical factors (HBV infection, hepatolithiasis, diabetes, cirrhosis, previous abdominal surgery, Child–Pugh classification, resection range, Charlson Comorbidity Index score, and anatomical resection). Anatomical resection (AR) is defined as resection of the tumor together with the portal veins draining the tumor and the corresponding hepatic territory, as determined by dye injection into the feeding portal vein. Non-anatomical resection (NAR) is defined as resection of a lesion regardless of the anatomical segment or section of the lobar anatomy and includes limited resection or enucleation. According to the accepted conferences in the literature (5, 9), minor LLR was regarded as a procedure in which ≤2 Couinaud segments located in the anterolateral part of the liver (II, III, IVb, V, VI) are removed. Major LLR was regarded as a procedure in which ≥3 segments are removed or involving the posterior superior segments (I, IVA, VII, VIII) regardless of the number of Couinaud segments removed. The long-term outcomes were overall survival (OS) and recurrence-free survival (RFS). The overall survival time was calculated from the day of operation to the time of death or the last follow-up. Recurrence-free survival was calculated from the day of surgery to the day of tumor recurrence or the last follow-up. The short-term outcomes included perioperative indicators, including blood loss, duration of surgery, intraoperative blood transfusion, postoperative blood transfusion, complications, duration of hospital stay, hospitalization expenses, and postoperative mortality. Complications were defined according to the Clavien–Dindo classification. Grade 1–2 complications were defined as minor complications and included wound infection (bedside), nausea, vomiting, and elevated blood pressure; grade 3 or higher complications were defined as major complications and included postoperative pleural effusion (excluding reactive pleural effusion in patients undergoing right liver resection), bile leakage, postoperative bleeding, liver failure, and death. Postoperative bile leakage and hepatocyte failure were defined by the international research group on liver surgery (18, 19). Postoperative mortality was defined as death occurring within 90 days after hepatectomy. The indications and contraindications of LLR are the same as those of OLR. This study was approved by the ethics committees of two centers. Early stage was defined as a unifocal lesion with a diameter of ≤3 cm and no vascular invasion, namely, Tis or T1a (≤3 cm) stage according to the AJCC eighth edition Cancer Staging system. **Figure 1** shows the study design.

### Statistical Analysis

Numerical variables are expressed as the mean ± SD or median (quartile range). χ^2^ or Fisher’s exact test was used to compare categorical variables, whereas the T test (normal distribution) and Wilcoxon rank sum test (nonnormal distribution) were used for continuous variables. The Kaplan–Meier method was used to obtain the cumulative survival rate. The log rank test was used to compare survival curves between the two groups. A two-tailed P<0.05 was considered to indicate significance. The nearest-neighbor matching method was used for the following matches. For the all-stage groups, the matching ratio was 1:1, and the caliper was 0.1. For the early-stage groups, the ratio was 1:2, and the caliper was 0.2. No samples with replacement was used. A stepwise backward Cox multivariable regression analysis including all available clinically relevant prognostic variables was used to identify prognostic factors for OS and RFS. Statistical analysis was carried out using R version 4.0.2 software for Windows.

## Results

A total of 1,084 patients with ICC were selected from the three centers, and data from 150 LLR patients and 645 OLR patients were obtained according to the above exclusion criteria. After matching, a total of 244 patients (122 in the LLR group and 122 in the OLR group) were included. The median follow-up was 33.2 months in both groups. **Table 1** summarizes the baseline of the variables before and after matching. Lymphatic invasion, vascular invasion, nerve invasion, differentiation, tumor size, BMI, AFP, CEA, CA19-9, previous abdominal surgery, ALB, MONO, Hb, and ASA grade showed significant differences between the groups before propensity score matching (PSM). Although the matching process does not completely eliminate all differences, these small differences are within the clinically acceptable range. **Table 2** summarizes the same baseline items between the two groups of early-stage patients, and the matching results are also acceptable. The median follow-up for early-stage patients was 26.0 (for OLR) and 31.2 (for LLR) months.

**Table 1:** Comparation of OLR and LLR groups in all-stage patients before and after matching.

	OLR N=645	LLR N=150	p	SMD	OLR N=122	LLR N=122	p	SMD
Sex			0.467	0.074			0.604	0.083
Female	312 (48.4%)	67 (44.7%)			49 (40.2%)	54 (44.3%)		
Male	333 (51.6%)	83 (55.3%)			73 (59.8%)	68 (55.7%)		
Age			0.427	0.081			0.213	0.178
≤65	405 (62.8%)	100 (66.7%)			89 (73.0%)	79 (64.8%)		
>65	240 (37.2%)	50 (33.3)			33 (27.0%)	43 (35.2%)		
BMI (kg/m^2^)	23.4 [20.8;25.6]	23.9 [21.8;26.0]	0.019	0.288	24.2 [21.4;27.2]	23.6 [21.7;25.8]	0.552	0.004
TNM:			0.165	0.216			0.988	0.002
Tis	3 (0.47%)	0 (0.00%)						
I	327 (50.7%)	79 (52.7%)			65 (53.3%)	66 (54.1%)		
II	84 (13.0%)	28 (18.7%)			25 (20.5%)	25 (20.5%)		
III	231 (35.8%)	43 (28.7%)			32 (26.2%)	31 (25.4%)		
Differentiation			0.022	0.25			0.669	0.115
Poorly differentiated/undifferentiated	240 (37.2%)	74 (49.3%)			60 (49.2%)	56 (45.9%)		
Moderately differentiated	324 (50.2%)	59 (39.3%)			52 (42.6%)	52 (42.6%)		
Well differentiated	81 (12.6%)	17 (11.3%)			10 (8.20%)	14 (11.5%)		
Lymphatic invasion			<0.001	0.56			0.767	0.076
No	510 (79.1%)	145 (96.7%)			115 (94.3%)	117 (95.9%)		
Yes	135 (20.9%)	5 (3.33%)			7 (5.74%)	5 (4.10%)		
Vascular invasion			<0.001	0.502			1	0.037
No	510 (79.1%)	143 (95.3%)			115 (94.3%)	116 (95.1%)		
Yes	135 (20.9%)	7 (4.67%)			7 (5.74%)	6 (4.92%)		
Nerve invasion			0.001	0.352			1	0.028
No	522 (80.9%)	139 (92.7%)			110 (90.2%)	111 (91.0%)		
Yes	123 (19.1%)	11 (7.33%)			12 (9.84%)	11 (9.02%)		
Tumor size	5.00 [3.50;7.00]	4.00 [3.00;6.00]	<0.001	0.358	5.00 [3.50;6.00]	4.35 [3.00;6.00]	0.267	0.076
Tumor number			0.086	0.16			0.863	0.044
1	573 (88.8%)	125 (83.3%)			103 (84.4%)	101 (82.8%)		
≥2	72 (11.2%)	25 (16.7%)			19 (15.6%)	21 (17.2%)		
HBV			0.252	0.111			0.189	0.186
No	459 (71.2%)	99 (66.0%)			69 (56.6%)	80 (65.6%)		
Yes	186 (28.8%)	51 (34.0%)			53 (43.4%)	42 (34.4%)		
Hepatolithiasis			0.095	0.164			1	<0.001
No	420 (65.1%)	109 (72.7%)			89 (73.0%)	89 (73.0%)		
Yes	225 (34.9%)	41 (27.3%)			33 (27.0%)	33 (27.0%)		
Diabetes			0.057	0.175			0.322	0.148
No	558 (86.5%)	120 (80.0%)			103 (84.4%)	96 (78.7%)		
Yes	87 (13.5%)	30 (20.0%)			19 (15.6%)	26 (21.3%)		
Hypertension			0.324	0.097			0.68	0.07
No	459 (71.2%)	100 (66.7%)			81 (66.4%)	85 (69.7%)		
Yes	186 (28.8%)	50 (33.3%)			41 (33.6%)	37 (30.3%)		
Fatty liver			1	0.035			0.684	0.106
No	624 (96.7%)	146 (97.3%)			120 (98.4%)	118 (96.7%)		
Yes	21 (3.26%)	4 (2.67%)			2 (1.64%)	4 (3.28%)		
Smoking			1	0.001			0.788	0.052
No	426 (66.0%)	99 (66.0%)			81 (66.4%)	78 (63.9%)		
Yes	219 (34.0%)	51 (34.0%)			41 (33.6%)	44 (36.1%)		
Drinking			0.105	0.16			0.89	0.035
No	435 (67.4%)	112 (74.7%)			83 (68.0%)	85 (69.7%)		
Yes	210 (32.6%)	38 (25.3%)			39 (32.0%)	37 (30.3%)		
Cirrhosis			0.21	0.121			1	0.02
No	534 (82.8%)	117 (78.0%)			95 (77.9%)	94 (77.0%)		
Yes	111 (17.2%)	33 (22.0%)			27 (22.1%)	28 (23.0%)		
Portal hypertension			0.597	0.079			1	<0.001
No	624 (96.7%)	147 (98.0%)			120 (98.4%)	120 (98.4%)		
Yes	21 (3.26%)	3 (2.00%)			2 (1.64%)	2 (1.64%)		
Ascites			0.397	0.099			0.11	0.241
No	600 (93.0%)	143 (95.3%)			111 (91.0%)	118 (96.7%)		
Yes	45 (6.98%)	7 (4.67%)			11 (9.02%)	4 (3.28%)		
Previous abdominal surgery			0.001	0.338			1	0.022
No	462 (71.6%)	128 (85.3%)			103 (84.4%)	102 (83.6%)		
Yes	183 (28.4%)	22 (14.7%)			19 (15.6%)	20 (16.4%)		
Child-Pugh classification			0.184	0.14			0.615	0.097
A	561 (87.0%)	137 (91.3%)			115 (94.3%)	112 (91.8%)		
B	84 (13.0%)	13 (8.67%)			7 (5.74%)	10 (8.20%)		
AFP (ng/ml)	3.60 [2.78;5.05]	3.14 [2.10;4.65]	<0.001	0.093	3.58 [2.88;5.04]	3.14 [2.10;4.62]	0.012	0.213
CEA (ng/ml)	3.20 [1.90;8.12]	2.88 [1.71;4.99]	0.038	0.244	3.12 [1.81;9.41]	2.84 [1.59;5.00]	0.122	0.268
CA19-9 (kU/L)	92.9 [21.8;670]	47.7 [16.4;452]	0.039	0.115	67.0 [19.0;557]	46.2 [16.7;405]	0.361	0.119
ALB (g/L)	39.7 [36.7;43.4]	41.7 [38.7;45.3]	<0.001	0.384	39.9 [37.6;44.2]	42.2 [39.3;45.3]	0.004	0.359
TBIL (μmol/L)	11.0 [7.70;16.7]	13.0 [9.22;16.9]	0.052	0.209	10.9 [7.40;14.5]	12.6 [9.16;16.6]	0.127	0.067
ALT (U/L)	22.0 [14.0;35.0]	22.0 [14.0;46.5]	0.476	0.05	21.0 [14.0;33.0]	21.0 [14.2;40.8]	0.412	0.049
AST (U/L)	27.0 [21.0;38.0]	25.0 [20.0;34.0]	0.084	0.096	24.5 [20.0;33.8]	24.0 [20.0;34.0]	0.801	0.059
NEU (×10^9^/L)	4.27 [3.17;5.67]	4.08 [3.01;5.65]	0.361	0.001	4.50 [3.05;5.73]	4.08 [3.03;5.37]	0.378	0.149
MONO (×10^9^/L)	0.51 [0.38;0.68]	0.47 [0.33;0.61]	0.047	0.161	0.56 [0.42;0.70]	0.48 [0.35;0.60]	0.001	0.258
LYM (×10^9^/L)	1.51 [1.18;1.90]	1.50 [1.15;2.11]	0.288	0.157	1.58 [1.28;1.99]	1.61 [1.20;2.11]	0.851	0.182
PLT (×10^9^/L)	210 [172;272]	212 [170;286]	0.871	0.028	220 [173;256]	214 [178;288]	0.435	0.053
Hb (g/L)	133 [120;144]	136 [123;148]	0.019	0.152	136 [125;148]	135 [124;148]	0.922	0.026
PT (s)	12.7 [11.6;13.6]	12.8 [11.8;13.6]	0.388	0.054	12.6 [11.9;13.5]	12.8 [11.8;13.6]	0.466	0.166
Chol (mmol/L)	4.57 [3.87;5.42]	4.56 [3.93;5.17]	0.308	0.211	4.31 [3.72;5.13]	4.61 [4.03;5.17]	0.072	0.04
TG (mmol/L)	1.15 [0.85;1.65]	1.24 [0.85;1.60]	0.825	0.08	1.10 [0.88;1.47]	1.25 [0.86;1.59]	0.287	0.028
HDL (mmol/L)	1.16 [0.93;1.37]	1.17 [0.95;1.41]	0.393	0.205	1.11 [0.93;1.27]	1.22 [0.96;1.42]	0.006	0.399
LDL (mmol/L)	2.68 [2.11;3.39]	2.55 [2.03;3.19]	0.187	0.168	2.58 [2.07;3.27]	2.70 [2.07;3.19]	0.684	0.004
ASA grade			<0.001	0.41			0.011	0.392
I	24 (3.72%)	20 (13.3%)			8 (6.56%)	12 (9.84%)		
II	576 (89.3%)	112 (74.7%)			111 (91.0%)	96 (78.7%)		
III	45 (6.98%)	18 (12.0%)			3 (2.46%)	14 (11.5%)		
Charlson Comorbidity Index score	5.00 [4.00;6.00]	5.00 [5.00;6.00]	<0.001	0.335	5.00 [4.00;6.00]	5.00 [5.00;6.00]	0.798	0.134
Resection range			0.023	0.215			0.701	0.066
Minor liver resection	288 (44.7%)	83 (55.3%)			62 (50.8%)	66 (54.1%)		
Major liver resection	357 (55.3%)	67 (44.7%)			60 (49.2%)	56 (45.9%)		
Anatomical resection			0.424	0.081			0.071	0.249
No	357 (55.3%)	77 (51.3%)			75 (61.5%)	60 (49.2%)		
Yes	288 (44.7%)	73 (48.7%)			47 (38.5%)	62 (50.8%)		

ALT, alanine aminotransferase; AST, aspartate aminotransferase; NEU, neutrophil; MONO, monocytes; LYM, lymphocyte; PLT, blood platelet; Hb, hemoglobin; PT, prothrombin time; Chol, cholesterol; TG, triglyceride; HDL, high-density lipoprotein; LDL, low-density lipoprotein.

**Table 2:** Comparation of OLR and LLR groups in early-stage patients before and after matching.

	OLR N=78	LLR N=32	p	SMD	OLR N=38	LLR N=27	p	SMD
Sex			0.053	0.449			0.339	0.309
Female	60 (76.9%)	18 (56.2%)			28 (73.7%)	16 (59.3%)		
Male	18 (23.1%)	14 (43.8%)			10 (26.3%)	11 (40.7%)		
Age			0.325	0.254			0.367	0.294
≤65	39 (50.0%)	20 (62.5%)			17 (44.7%)	16 (59.3%)		
>65	39 (50.0%)	12 (37.5%)			21 (55.3%)	11 (40.7%)		
BMI:			0.382	0.227			0.367	0.289
≤24	43 (55.1%)	14 (43.8%)			21 (55.3%)	11 (40.7%)		
>24	35 (44.9%)	18 (56.2%)			17 (44.7%)	16 (59.3%)		
Differentiation			0.012	0.625			1	0.079
Poorly differentiated / undifferentiated	15 (19.2%)	15 (46.9%)			14 (36.8%)	10 (37.0%)		
Moderately differentiated	48 (61.5%)	12 (37.5%)			18 (47.4%)	12 (44.4%)		
Well differentiated	15 (19.3%)	5 (15.6%)			6 (15.8%)	5 (18.5%)		
HBV			0.384	0.228			0.516	0.232
No	57 (73.1%)	20 (62.5%)			28 (73.7%)	17 (63.0%)		
Yes	21 (26.9%)	12 (37.5%)			10 (26.3%)	10 (37.0%)		
Hepatolithiasis			0.012	0.613			1	0.068
No	39 (50.0%)	25 (78.1%)			27 (71.1%)	20 (74.1%)		
Yes	39 (50.0%)	7 (21.9%)			11 (28.9%)	7 (25.9%)		
Diabetes			0.072	0.450			0.051	0.573
No	66 (84.6%)	22 (68.6%)			33 (86.8%)	17 (63.0%)		
Yes	12 (15.4%)	10 (31.4%)			5 (13.2%)	10 (37.0%)		
Cirrhosis			0.024	0.481			0.738	0.165
No	72 (92.3%)	24 (75.0%)			32 (84.2%)	21 (77.8%)		
Yes	6 (7.69%)	8 (25.0%)			6 (15.8%)	6 (22.2%)		
Hypertension:			0.764	0.108			0.676	0.173
No	48 (61.5%)	18 (56.2%)			27 (71.1%)	17 (63.0%)		
Yes	30 (38.5%)	14 (43.8%)			11 (28.9%)	10 (37.0%)		
Fattyl iver			0.083	0.365			0.329	0.400
No	78 (100%)	30 (93.8%)			38 (100%)	25 (92.6%)		
Yes	0 (0.00%)	2 (6.25%)			0 (0.00%)	2 (7.41%)		
Smoking			0.088	0.354			0.326	0.321
No	69 (88.5%)	24 (75.0%)			33 (86.8%)	20 (74.1%)		
Yes	9 (11.5%)	8 (25.0%)			5 (13.2%)	7 (25.9%)		
Drinking			0.776	0.083			0.571	0.226
No	66 (84.6%)	28 (87.5%)			29 (76.3%)	23 (85.2%)		
Yes	12 (15.4%)	4 (12.5%)			9 (23.7%)	4 (14.8%)		
Portal hypertension			0.671	0.203			0.586	0.268
No	72 (92.3%)	31 (96.9%)			34 (89.5%)	26 (96.3%)		
Yes	6 (7.69%)	1 (3.12%)			4 (10.5%)	1 (3.70%)		
Ascites			0.555	0.283			0.630	0.333
No	75 (96.2%)	32 (100%)			36 (94.7%)	27 (100%)		
Yes	3 (3.85%)	0 (0.00%)			2 (5.26%)	0 (0.00%)		
Previous abdominal surgery			0.005	0.725			0.026	0.699
No	48 (61.5%)	29 (90.6%)			25 (65.8%)	25 (92.6%)		
Yes	30 (38.5%)	3 (9.38%)			13 (34.2%)	2 (7.41%)		
Child-Pugh classification			0.276	0.327			0.388	0.345
A	69 (88.5%)	31 (96.9%)			33 (86.8%)	26 (96.3%)		
B	9 (11.5%)	1 (3.12%)			5 (13.2%)	1 (3.70%)		
AFP (ng/ml)	3.19 [2.48;4.36]	3.55 [2.42;6.95]	0.133	0.475	3.35 [2.16; 4.22]	3.54 [2.68; 7.61]	0.636	0.383
CEA(ng/ml)			0.756	0.116			0.583	0.209
≤5	57 (73.1%)	25 (78.1%)			26 (68.4%)	21 (77.8%)		
>5	21 (26.9%)	7 (21.9%)			12 (31.6%)	6 (22.2%)		
CA19-9 (kU/L)			0.077	0.429			0.828	0.123
≤37	40 (51.3%)	23 (71.9%)			26 (68.4%)	20 (74.1%)		
>37	38 (48.7%)	9 (28.1%)			12 (31.6%)	7 (25.9%)		
ALB (g/L)	39.5 [35.4;42.4]	42.2 [40.3;44.8]	0.002	0.665	38.8 [35.0;42.6]	42.0 [40.2;44.2]	0.015	0.670
TBIL (μmol/L)	10.6 [7.00;14.0]	12.0 [9.60;16.0]	0.151	0.297	12.2 [9.48;22.6]	11.3 [9.50;15.3]	0.452	0.514
ALT (U/L)	22.5 [16.0;35.0]	26.5 [21.8;42.5]	0.431	0.290	21.0 [17.0;34.0]	24.0 [21.0;40.0]	0.398	0.206
AST (U/L)	25.0 [20.2;33.0]	28.0 [19.8;35.0]	0.971	0.107	24.5 [20.0;31.8]	28.0 [19.0;33.0]	0.931	0.101
NEU (×10^9^/L)	3.82 [2.91;4.81]	3.05 [2.23;3.90]	0.011	0.594	4.18 [2.94;5.03]	3.03 [2.10;3.73]	0.008	0.733
MONO (×10^9^/L)	0.48 [0.34;0.64]	0.32 [0.26;0.39]	<0.001	0.438	0.54 [0.39;0.71]	0.32 [0.26;0.40]	<0.001	1.045
LYM (×10^9^/L)	1.44 [1.25;1.80]	1.38 [0.87;1.85]	0.483	0.273	1.44 [1.25;1.72]	1.39 [0.95;1.90]	0.973	0.088
PLT (×10^9^/L)	198 [156;237]	180 [166;214]	0.359	0.376	203 [162;290]	180 [168;214]	0.239	0.610
Hb (g/L)	130 [119;140]	140 [126;158]	0.009	0.530	130 [120;141]	137 [122;154]	0.156	0.360
PT (s)	12.9 [11.8;13.5]	11.8 [11.3;13.6]	0.298	0.210	12.8 [11.8;13.5]	11.7 [11.3;13.4]	0.141	0.203
Chol (mmol/L)	4.74 [3.97;5.58]	4.78 [4.08;5.19]	0.403	0.359	5.00 [4.24;5.72]	4.79 [4.36;5.23]	0.208	0.415
TG (mmol/L)	1.14 [0.89;1.65]	1.50 [0.96;1.63]	0.33	0.104	1.18 [0.98;1.48]	1.53 [1.02;1.64]	0.348	0.202
HDL (mmol/L)	1.21 [1.03;1.39]	1.16 [0.91;1.25]	0.225	0.435	1.33 [1.17;1.40]	1.14 [0.95;1.26]	0.004	0.786
LDL (mmol/L)	2.62 [2.22;3.11]	2.66 [2.06;3.33]	0.688	0.166	2.70 [2.13;3.42]	2.72 [2.13;3.35]	0.968	0.123
ASA grade			<0.001	0.819			<0.001	1.000
I	3 (3.85%)	3 (9.38%)			0 (0.00%)	2 (7.41%)		
II	75 (96.2%)	22 (68.8%)			38 (100%)	18 (66.7%)		
III	0 (0.00%)	7 (21.9%)			0 (0.00%)	7 (25.9%)		
Charlson Comorbidity Index score			0.706	0.123			1	0.025
≤5	51 (65.4%)	19 (59.4%)			23 (60.5%)	16 (59.3%)		
>5	27 (34.6%)	13 (40.6%)			15 (39.5%)	11 (40.7%)		
Resection range			0.599	0.153			1	0.015
Minor liver resection	45 (57.7%)	16 (50.0%)			18 (47.4%)	13 (48.1%)		
Major liver resection	33 (42.3%)	16 (50.0%)			20 (52.6%)	14 (51.9%)		
Hospitalization expenses	55,418 [46,011;67,247]	51,747 [36,570;71,556]	0.392	0.148	62,201 [48,431;70,445]	55,575 [38,448;72,508]	0.293	0.263
Anatomical resection			0.41	0.215			0.488	0.236
No	45 (57.7%)	15 (46.9%)			20 (52.6%)	11 (40.7%)		
Yes	33 (42.3%)	17 (53.1%)			18 (47.4%)	16 (59.3%)		

**Table 3** summarizes the perioperative results of the two groups of all-stage patients. There were significant differences observed in blood loss [blood loss >400 ml 27 (22.1%) for OLR *vs.* 6 (4.92%) for LLR, p<0.001], the duration of hospital stay (11.0 [9.00;16.0] for OLR *vs.* 9.00 [7.00;12.0] for LLR, p<0.001), and severe complications [18 (14.8%) for OLR *vs.* 7 (5.74%) for LLR, p=0.032]. **Table 4** shows the perioperative results of the early-stage patients. Similar to the all-stage patients, the LLR group had less blood loss [blood loss >400 ml 12 (31.6%) for OLR *vs.* 2 (7.41%) for LLR, p=0.042] and a shorter duration of hospital stay [11.0 (8.50;17.8) for OLR *vs.* 9.00 (6.50;11.0) for LLR, p=0.011] than the OLR group. In addition, the LLR group had a shorter duration of surgery [200 (141;249) min for OLR *vs.* 125 (115;222) min for LLR, p=0.025].

**Table 3:** Comparation of short-term outcomes between the two groups before and after matching in all-stage patients.

	OLR N=122	LLR N=122	p
Duration of surgery (min)	168 [120;210]	170 [130;240]	0.087
Duration of hospital stay (days)	11.0 [9.00;16.0]	9.00 [7.00;12.0]	<0.001
Hospitalization expenses	55,594 [44,720;70,444]	56,693 [43,539;70,138]	0.91
Blood loss (ml)			<0.001
≤400	95 (77.9%)	116 (95.1%)	
>400	27 (22.1%)	6 (4.92%)	
Intraoperative blood transfusion			0.146
No	105 (86.1%)	113 (92.6%)	
Yes	17 (13.9%)	9 (7.38%)	
Postoperative blood transfusion			0.683
No	110 (90.2%)	107 (87.7%)	
Yes	12 (9.84%)	15 (12.3%)	
Clavien-Dindo classification			0.032
≤2	104 (85.2%)	115 (94.3%)	
≥3	18 (14.8%)	7 (5.74%)	
Mortality			1
No	118 (96.7%)	118 (96.7%)	
Yes	4 (3.28%)	4 (3.28%)	

**Table 4:** Comparation of short-term outcomes between the two groups before and after matching in early-stage patients.

	OLR N=38	LLR N=27	p
Duration of surgery (min)	200 [141;249]	125 [115;222]	0.025
Duration of hospital stay (days)	11.0 [8.50;17.8]	9.00 [6.50;11.0]	0.011
Hospitalization expenses	62,201 [48,431;70,445]	55,575 [38,448;72,508]	0.293
Blood loss (ml)			0.042
≤400	26 (68.4%)	25 (92.6%)	
>400	12 (31.6%)	2 (7.41%)	
Intraoperative blood transfusion			0.169
No	38 (100%)	25 (92.6%)	
Yes	0 (0.00%)	2 (7.41%)	
Postoperative blood transfusion			0.169
No	38 (100%)	25 (92.6%)	
Yes	0 (0.00%)	2 (7.41%)	
Clavien-Dindo classification			0.636
≤2	35 (92.1%)	26 (96.3%)	
≥3	3 (7.89%)	1 (3.70%)	
Mortality			1
No	38 (100%)	27 (100%)	
Yes	0	0	

**Figure 2:**
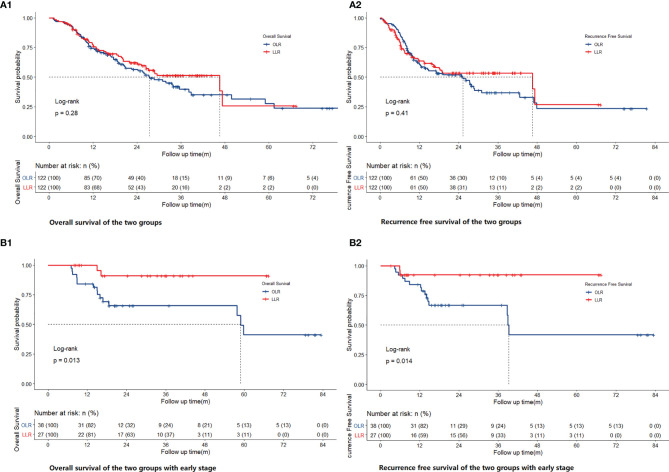
Comparison of OS [**(A1)** for all stage, **(B1)** for early stage] and RFS [**(A2)** for all stage, **(B2)** for early stage] of OLR and LLR after PSM.

**Figures 2A1, A2** show the overall survival (OS) and recurrence-free survival (RFS) of the two groups of all-stage patients after PSM, and **Supplementary 1** shows the same items before PSM. Different from the trend before matching (p=0.0013 for OS, p=0.0019 for RFS), no significant differences were found for all-stage patients (p=0.28 for OS, p=0.41 for RFS) after PSM. For early-stage patients, there were significant differences before (p=0.0014 for OS, p=0.0028 for RFS) (**Supplementary 1**) and after matching (p=0.013 for OS, p=0.014 for RFS) (**Figures 2B1, B2**). After matching, the 1-, 3-, and 5-year OS rates of the OLR group of all-stage patients were 74.4, 39.8, and 27.6%, respectively, and those of the LLR group were 77.3, 51.4, and 25.7%, respectively. The RFS rates of the OLR group were 60.6, 36.9, and 23.4%, respectively, and those of the LLR group were and 63.7, 53.5, and 26.7% (RFS), respectively. Correspondingly, the 1-, 3-, and 5-year OS rates of the OLR group of early-stage patients were 84.2, 65.8, and 41.1%, and the RFS rates were 84.2, 66.7, and 41.7%, respectively. The OS and RFS rates of the LLR group were 100%, 90.9%, and 90.9% and 92.3, 92.3, and 92.3%, respectively (**Supplementary 2**).

For all-stage patients, TNM stage, differentiation, tumor size, HBV infection, hepatolithiasis, postoperative blood transfusion, resection range, and comorbidity were independent prognostic factors for OS. TNM stage, HBV infection, hepatolithiasis, resection range, and postoperative blood transfusion were independent prognostic factors for RFS. For early-stage patients, CEA >5 ng/ml and blood loss >400 ml were independent prognostic factors for OS. Hepatolithiasis, CEA >5 ng/ml, blood loss >400 ml, and Child–Pugh classification were independent prognostic factors for RFS (**Supplementary 3**).

## Discussion

The widespread use of laparoscopic hepatectomy stands in stark contrast to its small-scale application in ICC. This may be related to the following factors. On the one hand, ICC has a very low incidence rate, comprising approximately 1.5~3% of all primary liver tumors (20). Even the increasing incidence rate in recent years (21) cannot compensate for the small total number. On the other hand, most ICC patients lose the opportunity for surgery at the time of the initial diagnosis because of its asymptomatic nature. An analysis of the data of ICC patients in the SEER database from 1983 to 2010 revealed that only approximately 12.5% of the patients underwent surgical treatment (22). Third, patients often need major liver resection because of the unique oncological characteristics of ICC. This means the reconstruction of large vessels and bile ducts, as well as adequate lymph node dissection, increases the difficulty of laparoscopic resection. Studies have shown that 50–70% of resectable ICC patients undergo hemihepatectomy or extended hepatectomy (23–25).

Despite the above limitations, based on the reported ICC cases, it has been concluded that laparoscopic treatment is no less effective than open hepatectomy (14–16). However, comparative studies of LLR and OLR in early-stage ICC are rare, so the benefits of minimally invasive surgery for early-stage patients remain unclear. Propensity score matching (PSM) has been favored by researchers in recent years. Despite the controversies regarding the selection of variables for generating propensity score models, some scholars have suggested that potential confounding variables not related to exposure but related to the results should be included in the propensity score model (26).

**Figure 3:**
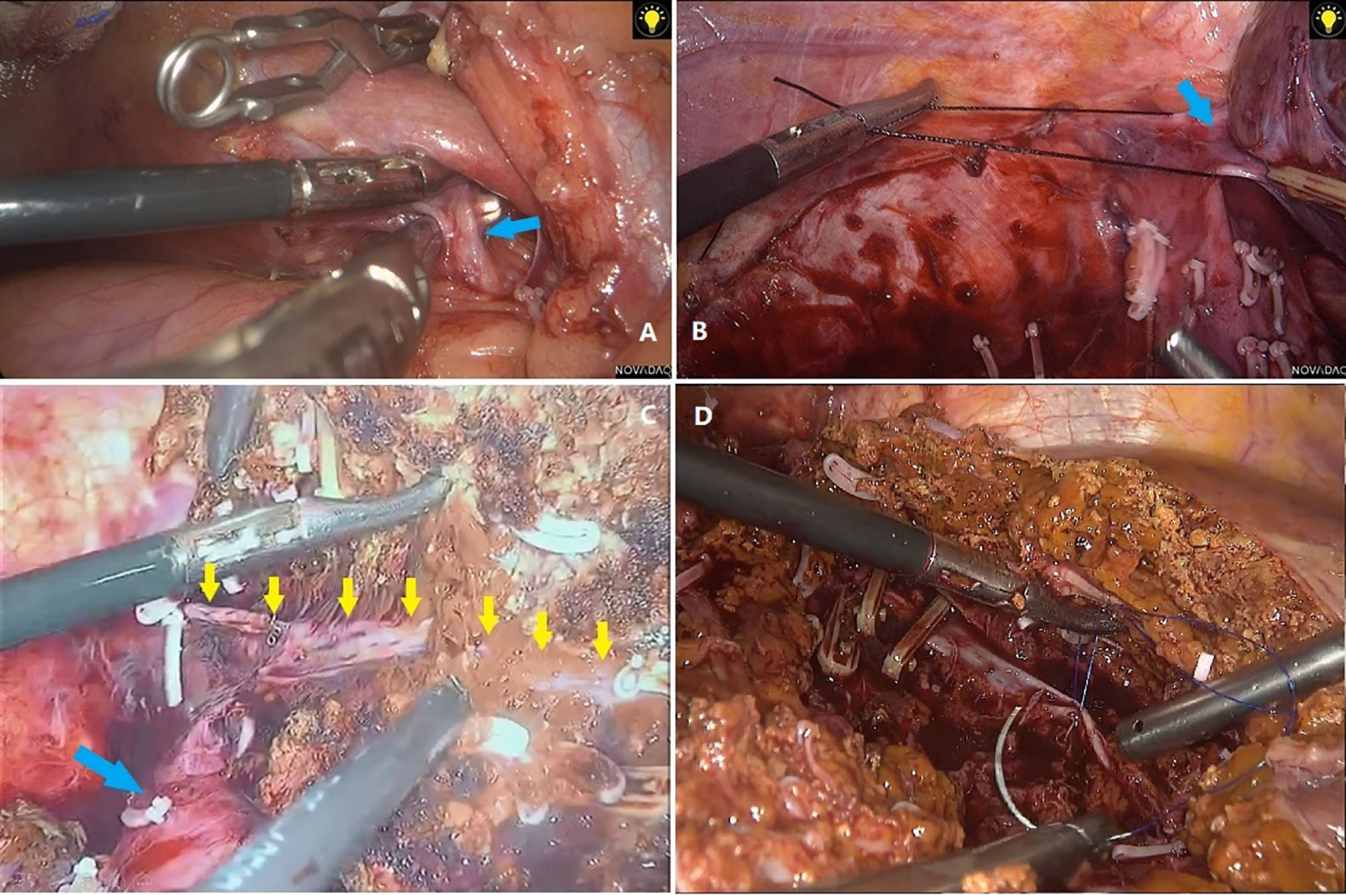
**(A)** Separation of short hepatic veins with right angle forceps. **(B)** Dissociation of right hepatic vein. **(C)** Anatomical resection showed right hepatic vein (yellow arrows) and inferior vena cava (blue arrow). **(D)** Suture of bleeding hepatic veins.

After matching, based on the short-term results, the LLR group had less blood loss (p=0.001 in all-stage, p=0.042 in early-stage) and a shorter hospital stay (p<0.001 in all-stage, p=0.011 in early-stage) than the OLR group, regardless of whether all-stage or early-stage patients were analyzed. The lower blood loss in the LLR group indicates that skilled vascular management may be key to the successful implementation of LLR. The three aspects of vascular management can be summarized as follows: The first point is to control hepatic blood inflow. The main methods at present are the Pringle maneuver and regional blood flow occlusion of tumor-bearing liver segments. The second aspect is the control of blood outflow, especially for patients with tumors located in I, VII, VIII, IVa, or right hemihepatectomy. Dissecting and exposing the hepatic veins of the second hepatic portal (**Figure 3B**) and the short hepatic vessels of the third hepatic portal (**Figure 3A**) are very important for safe resection and to reduce bleeding. High-definition magnified laparoscopic images provide a more precise perspective for managing “difficult” hepatic vessels, while the application of some tools (such as “golden finger,” right angle forceps, etc.) (**Figure 3A**) makes the process of vascular disconnection safer and simpler. The third point is the management of accidental bleeding. Skill in the laparoscopic suture technique is key to avoiding unnecessary conversion (**Figure 3D**). In many cases, large blood vessels are regarded as a “forbidden zone,” but the actual situation is not “the farther away, the better.” If the structures around the large vessels are not dissected clearly, it can be difficult to suture under laparoscopy or after conversion to open surgery because the large vessels are ruptured. Thus, we suggest that dissociating the perivascular structure and clamping it safely should be done first to prevent possible accidental bleeding. The incidence of complications is also an important indicator of surgical quality and is also related to the duration of hospital stay. For all-stage patients, the incidence of severe complications (grade III and above) in the LLR group was lower (p=0.032) than that in the OLR group, which is also consistent with the results of previous studies on laparoscopic hepatectomy (1). This may be one of the reasons for the shorter hospital stay, but no similar difference was found in the comparison of patients with early-stage ICC (p=0.636). Therefore, the shorter hospital stay may be attributed to the lighter trauma burden of minimally invasive technology, not just complications. However, no difference in hospitalization costs was seen despite the difference in hospital stay duration (p=0.91 for all stage, p=0. 0.293 for early stage).

Relatively speaking, major resections are more often performed in ICC patients and can cause excessive blood loss, the need for blood transfusion, severe complications, and a longer surgery compared with minor resection (5, 7). In this study, major LLR included right hemihepatectomy, right posterior lobectomy, left half + left caudate lobe, right half + partial caudate lobe, middle hepatectomy, and other resections involving segments IVA, VII, VIII, and I. This process requires anatomical or non-anatomical resection, which depends on the volume of the remnant liver or lesion location (**Figure 3C**).

Surgical experience is also an important factor affecting short-term and long-term outcomes. A lack of experience with certain techniques may have disastrous oncological consequences for ICC patients with a high metastatic burden. The recent European consensus suggested that the learning curve for minor resections is 60 cases, while that for major resections is 55 cases on the basis of minor resections (10). Therefore, the years of experience (≥6 years) and the cumulative number of LLR cases (≥60 cases) should be considered, and patients who did not meet these criteria were excluded (**Figure 1**).

For the long-term outcomes, no significant difference was found in the all-stage groups after PSM (p=0.28 for OS, p=0.41 for RFS). This conclusion is consistent with that of recent studies on the same subject (15, 17, 27). In fact, most of the current studies on HCC have not found significant differences in long-term oncological outcomes between laparoscopic and open approaches. However, based on the long-term outcomes of the early-stage groups, significant differences were found between the two groups before (p=0.0014 for OS, p=0.0028 for RFS) and after matching (p=0.013 for OS, p=0.014 for RFS). This may be due to the oncological benefit of laparoscopy. As mentioned above, high-definition surgical images, the convenience and safety of laparoscopic lenses and instruments when dealing with “difficult” posterosuperior segments are all possible reasons. Another possible reason is that surgery itself profoundly suppresses cell-mediated immunity (CMI) (28). The exaggerated and prolonged inflammatory, metabolic, and catabolic responses caused by major surgery induce clinical complications, delay recovery, increase mortality (29), and promote cancer metastasis (30–32). Some studies have also confirmed that surgical stress has a negative impact on long-term survival outcomes in patients with colorectal cancer (33). Under the same conditions, we consider that the trauma incurred from laparoscopy is milder than that incurred from open surgery, which also provides a possible explanation to promote this approach for patients with cholangiocarcinoma. In addition, it is not easy to assess whether there are differences in long-term survival outcomes due to anthropic factors. For example, we speculate that if the preoperative assessment indicates that the tumor is adjacent to larger vessels, some surgeons may prefer open surgery; otherwise, laparoscopic surgery may be considered. Therefore, as ICC is more invasive than HCC in terms of its oncological characteristics, it is necessary to be cautious when making conclusions regarding tumor differences or the equivalence of laparoscopic treatment for ICC. Preoperative imaging may not be able to accurately distinguish some ICCs from HCC in clinical practice, but in view of the benefits of laparoscopic treatment compared with open surgery (34, 35) and the similar results of this retrospective study for patients with early HCC, the clinical significance of this study is that laparoscopic surgery can be recommended for patients with a tumor diameter of ≤3 cm and no vascular invasion—even if it is difficult to distinguish ICC from HCC on imaging. Higher-quality studies should be carried out to further verify the role of laparoscopy in early ICC patients, which may have a certain impact on the choice of surgical methods.

## Conclusion

Patients treated with laparoscopy seem to have better short-term outcomes, such as less blood loss, shorter operation duration, and shorter hospital stay, than patients undergoing open surgery. Based on the long-term results, no significant difference was found between OS and RFS in all-stage patients, but the differences were more obvious in early-stage patients. Future research needs to examine the outcomes of early-stage ICC patients from different aspects, such as long-term complications, and focus on the improving the quality of these patients’ lives.

## Data Availability Statement

The original contributions presented in the study are included in the article/**Supplementary Material**. Further inquiries can be directed to the corresponding authors.

## Ethics Statement

The studies involving human participants were reviewed and approved by Ethics Committee in Clinical Research of the First Affiliated Hospital of Wenzhou Medical University. The data used in this article are all items that must be checked according to medical standards during the hospitalization, and collected retrospectively when designing the study, without adding any additional medical examination or test outside the normal diagnosis and treatment procedures.

## Author Contributions

YJ is responsible for the conception, design, and writing of the article. WYi and ZY are responsible for the data processing and analysis. MD, CX, WY, DL, YH, WL, DT, CK, HJ, ZC, and WD are responsible for collecting the original data. JB and CG are responsible for reviewing and guiding the revision of the paper. All authors contributed to the article and approved the submitted version.

## Funding

This study was supported by the National Natural Science Foundation of China (81772628, 81703310, 82072685), the Research Foundation of National Health Commission of China–Major Medical and Health Technology Project for Zhejiang Province (WKJ-ZJ-1706).

## Conflict of Interest

The authors declare that the research was conducted in the absence of any commercial or financial relationships that could be construed as a potential conflict of interest.

## Publisher’s Note

All claims expressed in this article are solely those of the authors and do not necessarily represent those of their affiliated organizations, or those of the publisher, the editors and the reviewers. Any product that may be evaluated in this article, or claim that may be made by its manufacturer, is not guaranteed or endorsed by the publisher.
