# Supplementary figures and images for: Laparoscopic Versus Open Surgery for Early-Stage Intrahepatic Cholangiocarcinoma After Mastering the Learning Curve: A Multicenter Data-Based Matched Study
Source: Front Oncol. 2022 Jan 7;11:742544. doi: 10.3389/fonc.2021.742544 (PMC8777042; doi:10.3389/fonc.2021.742544)

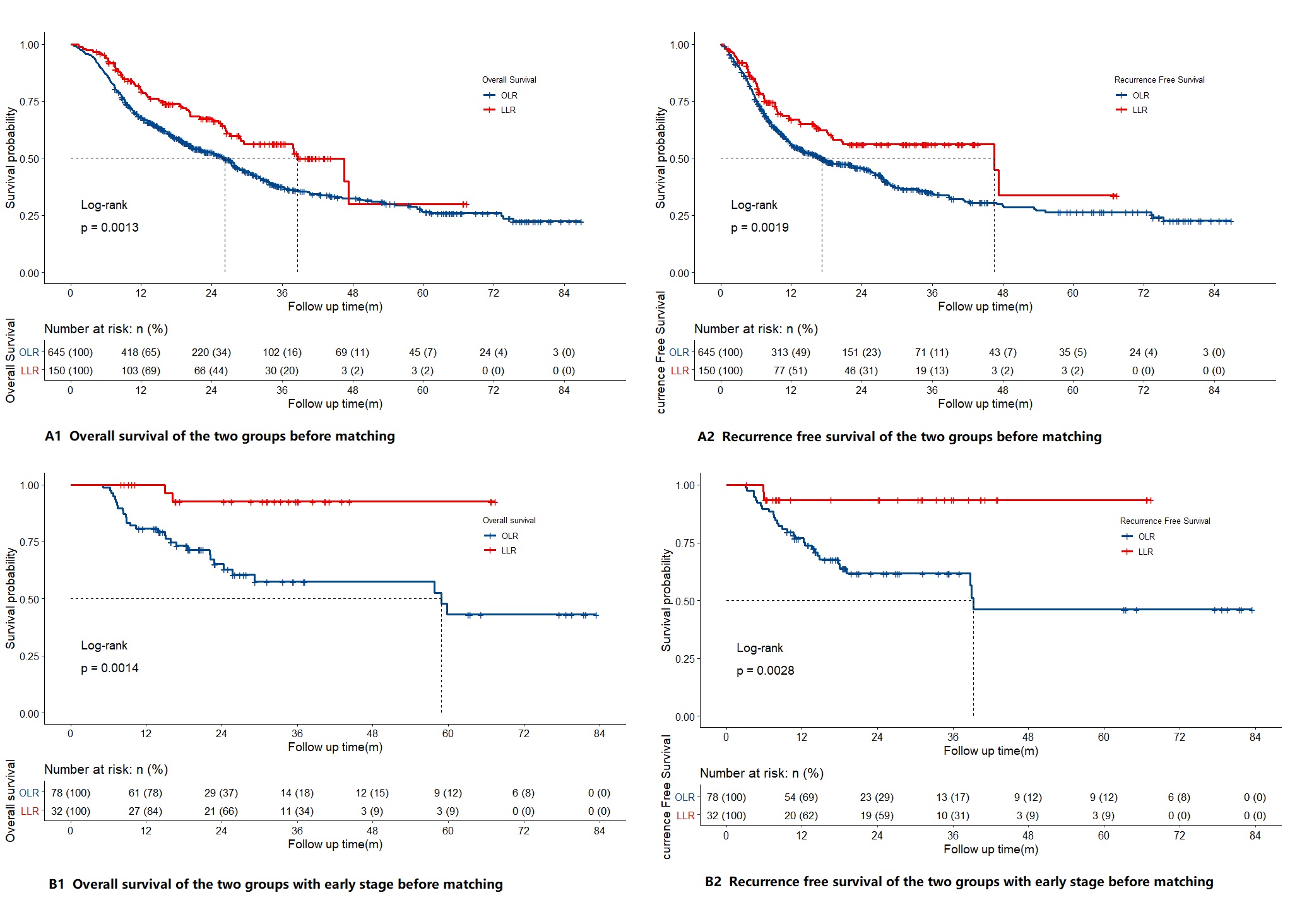

Supplement: Supplementary file 1 [file Image_1.jpeg]
