# Supplementary material for: Laparoscopic Versus Open Surgery for Early-Stage Intrahepatic Cholangiocarcinoma After Mastering the Learning Curve: A Multicenter Data-Based Matched Study
Source: Front Oncol. 2022 Jan 7;11:742544. doi: 10.3389/fonc.2021.742544 (PMC8777042; doi:10.3389/fonc.2021.742544)
Supplement: Supplementary file 2 [file Table_1.docx]

Supplementary 2 The overall survival and recurrence free survival between the two groups

| After PSM | All stage | | | Early stage | | |
| --- | --- | --- | --- | --- | --- | --- |
|  | 1 year | 3 year | 5 year | 1 year | 3 year | 5 year |
| **OLR** |  |  |  |  |  |  |
| Overall Survival (%) | 74.4 | 39.8 | 27.6 | 84.2 | 65.8 | 41.1 |
| Recurrence Free Survival (%) | 60.6 | 36.9 | 23.4 | 84.2 | 66.7 | 41.7 |
| **LLR** |  |  |  |  |  |  |
| Overall Survival (%) | 77.3 | 51.4 | 25.7 | 100 | 90.9 | 90.9 |
| Recurrence Free Survival (%) | 63.7 | 53.5 | 26.7 | 92.3 | 92.3 | 92.3 |

| Before PSM | All stage | | | Early stage | | |
| --- | --- | --- | --- | --- | --- | --- |
|  | 1 year | 3 year | 5 year | 1 year | 3 year | 5 year |
| **OLR** |  |  |  |  |  |  |
| Overall Survival (%) | 67.8 | 37.3 | 26.5 | 80.8 | 56.4 | 42.3 |
| Recurrence Free Survival (%) | 56.0 | 34.6 | 26.4 | 76.8 | 61.6 | 46.2 |
| **LLR** |  |  |  |  |  |  |
| Overall Survival (%) | 80.1 | 56.3 | 29.9 | 100 | 92.6 | 92.6 |
| Recurrence Free Survival (%) | 66.8 | 56.1 | 33.7 | 93.5 | 93.5 | 93.5 |
