# Supplementary material for: Laparoscopic Versus Open Surgery for Early-Stage Intrahepatic Cholangiocarcinoma After Mastering the Learning Curve: A Multicenter Data-Based Matched Study
Source: Front Oncol. 2022 Jan 7;11:742544. doi: 10.3389/fonc.2021.742544 (PMC8777042; doi:10.3389/fonc.2021.742544)
Supplement: Supplementary file 3 [file Table_2.docx]

Supplementary 3 Cox regression analyses of prognostic factors associated with Overall Survival and Recurrence Free Survival

All-stage

|  | **Hazard Ratio** | **P** |
| --- | --- | --- |
| **Overall Survival** |  |  |
| TNM stage |  |  |
| 1 | 1 (Ref) |  |
| 2 | 0.59 (0.31-1.12) | 0.107 |
| 3 | 2.12 (1.31-3.43) | 0.022 |
| Differentiation |  |  |
| Poor | 1 (Ref) |  |
| Moderate | 0.78 (0.52-1.18) | 0.24 |
| Well | 0.25 (0.11-0.59) | 0.001 |
| Tumor size (per 1-cm increase) | 1.07 (1.01-1.15) | 0.033 |
| HBV |  |  |
| No | 1 (Ref) |  |
| Yes | 0.53 (0.34-0.83)( | 0.005 |
| Hepatolithiasis |  |  |
| No | 1 (Ref) |  |
| Yes | 2.93 (1.91-4.49) | ＜0.001 |
| Resection range |  |  |
| Minor | 1 (Ref) |  |
| Major | 1.67 (1.09-2.57) | 0.019 |
| Postoperative blood transfusion |  |  |
| No | 1 (Ref) |  |
| Yes | 0.35 (0.16-0.75) | 0.007 |
| Charlson Comorbidity Index score (per 1-score increase) | 1.18 (1.04-1.33) | 0.009 |
| **Recurrence Free Survival** |  |  |
| TNM stage |  |  |
| 1 | 1 (Ref) |  |
| 2 | 0.63 (0.34-1.18) | 0.147 |
| 3 | 2.02 (1.26-3.24) | 0.004 |
| HBV |  |  |
| No | 1 (Ref) |  |
| Yes | 0.49 (0.32-0.75) | 0.001 |
| Hepatolithiasis |  |  |
| No | 1 (Ref) |  |
| Yes | 3.15 (2.08-4.79) | ＜0.001 |
| Resection range |  |  |
| Minor | 1 (Ref) |  |
| Major | 1.93 (1.26-2.96) | 0.003 |
| Postoperative blood transfusion |  |  |
| No | 1 (Ref) |  |
| Yes | 0.33 (0.15-0.72) | 0.005 |
| Charlson Comorbidity Index score (per 1-score increase) | 1.07 (0.95-1.21) | 0.26 |

Early-stage

|  | Hazard Ratio | P |
| --- | --- | --- |
| **Overall Survival** |  |  |
| Hepatolithiasis |  |  |
| No | 1 (Ref) |  |
| Yes | 2.7（0.91-8.1） | 0.072 |
| CEA (ng/ml) |  |  |
| ≤5 | 1 (Ref) |  |
| ＞5 | 3.4（1.35-8.4） | 0.009 |
| Ascites |  |  |
| No | 1 (Ref) |  |
| Yes | 5.4（0.66-43.5） | 0.115 |
| Child-Pugh classification |  |  |
| A | 1 (Ref) |  |
| B | 2.5（0.83-7.5） | 0.102 |
| Blood loss（ml） |  |  |
| ≤400 | 1 (Ref) |  |
| ＞400 | 4.8（1.84-12.3） | 0.001 |
| Anatomical resection |  |  |
| No | 1 (Ref) |  |
| Yes | 1.9（0.77-4.6） | 0.168 |
| **Recurrence Free Survival** |  |  |
| Hepatolithiasis |  |  |
| No | 1 (Ref) |  |
| Yes | 3.5 （1.24-9.9） | 0.018 |
| CEA (ng/ml) |  |  |
| ≤5 | 1 (Ref) |  |
| ＞5 | 2.88（1.2-6.9） | 0.018 |
| Ascites |  |  |
| No | 1 (Ref) |  |
| Yes | 5.74（0.84-39.2） | 0.075 |
| Child-Pugh classification |  |  |
| A | 1 (Ref) |  |
| B | 3.10（1.09-8.8） | 0.034 |
| Blood loss（ml） |  |  |
| ≤400 | 1 (Ref) |  |
| ＞400 | 3.04（1.31-7.0） | 0.01 |
